# The first darter (Aves: Anhingidae) fossils from India (late Pliocene)

**DOI:** 10.1371/journal.pone.0177129

**Published:** 2017-05-24

**Authors:** Thomas Stidham, Rajeev Patnaik, Kewal Krishan, Bahadur Singh, Abhik Ghosh, Ankita Singla, Simran S. Kotla

**Affiliations:** 1 Key Laboratory of Vertebrate Evolution and Human Origins of the Chinese Academy of Sciences, Institute of Vertebrate Paleontology and Paleoanthropology, Chinese Academy of Sciences, Beijing, China; 2 Department of Geology, Panjab University, Chandigarh, India; 3 Department of Anthropology, Panjab University, Chandigarh, India; Royal Belgian Institute of Natural Sciences, BELGIUM

## Abstract

New fossils from the latest Pliocene portion of the Tatrot Formation exposed in the Siwalik Hills of northern India represent the first fossil record of a darter (Anhingidae) from India. The darter fossils possibly represent a new species, but the limited information on the fossil record of this group restricts their taxonomic allocation. The Pliocene darter has a deep pit on the distal face of metatarsal trochlea IV not reported in other anhingids, it has an open groove for the m. flexor perforatus et perforans digiti II tendon on the hypotarsus unlike New World anhingid taxa, and these darter specimens are the youngest of the handful of Neogene records of the group from Asia. These fossil specimens begin to fill in a significant geographic and temporal gap in the fossil record of this group that is largely known from other continents and other time periods. The presence of a darter and pelican (along with crabs, fish, turtles, and crocodilians) in the same fossil-bearing horizon strongly indicates the past presence of a substantial water body (large pond, lake, or river) in the interior of northern India in the foothills of the Himalayan Mountains.

## Introduction

While the first fossil birds from India were published in the 19^th^ century [1,2], the known diversity of birds in India has not expanded greatly in the intervening century. The fossil record of birds in India now includes several Eocene taxa (e.g., [3]), the Neogene taxa from the Siwaliks Hills (e.g., [1, 4]), and some Pleistocene material (e.g., [5]). Overall, the known past diversity of birds in India is not rich, and the fossil record of birds across southern Asia is generally poorly known (e.g., [4,6,7]). Neogene fossils of birds from India and neighboring Pakistan include pelicans, storks, ostriches, and other terrestrial and aquatic taxa [1,2,4,8].

**Fig 1:**
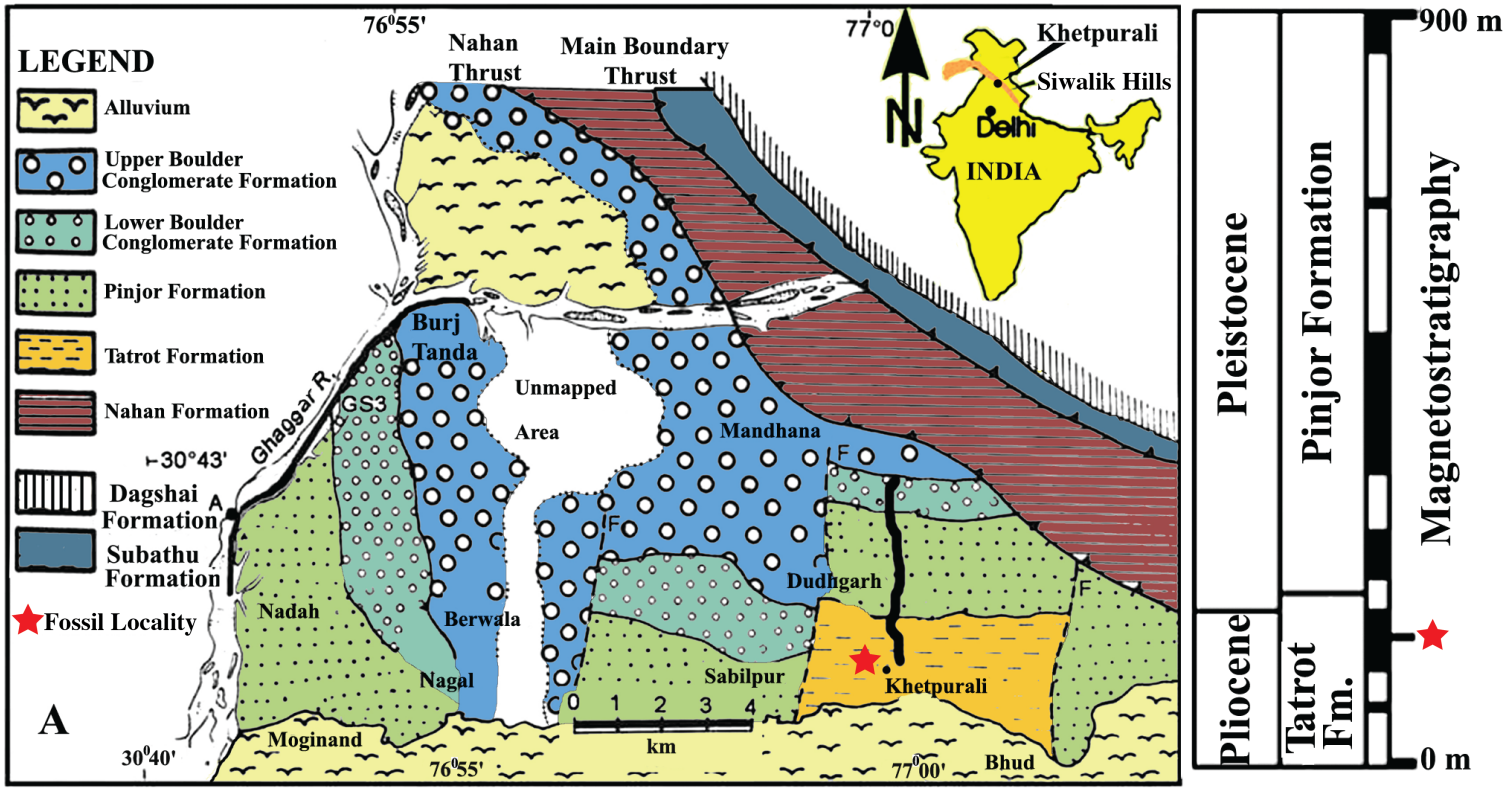
The geology, location, stratigraphy, and magnetostratigraphy of the Ketpurali section and region, northern India. The correlation of the lithostratigraphy with the magnetostratigraphy (and geochronological boundaries) is provided on the right side. The vertebrate fossil bone horizon is marked with a red star (map and stratigraphic section). The figure is modified from [4].

Recent fieldwork by individuals at Panjab University (India) is adding new taxa and additional specimens to the Indian Neogene record from the Siwalik Hills in northern India (e.g., [4] and the new fossils below; Fig 1). Further exploration of the stratigraphic sequence and locality (Fig 1) that produced the recently reported pelican specimen [4] continues to result in the discovery of new avian fossils. This fossil horizon in the Khetpurali section in the upper part of the Tatrot Formation (extending ~5.26 to ~2.58 Ma, see [4] for a longer discussion of the stratigraphy and geology) is from within the last normal polarity magnetochron of the Pliocene, and is approximately 2.6 Ma (Fig 1). Among those newly located specimens are the first records of a darter (Anhingidae; commonly called darters, anhingas, or snake birds) reported below. All three of the new skeletal specimens are fragments of tarsometatarsi, and our aim is to phylogenetically constrain the identification of these fossils, discuss their evolutionary and biogeographic importance, and ascertain the morphological variation among members in that taxonomic group. Today one species of darter (*Anhinga melanogaster*) occurs in across much of India, excluding the Himalayas and Northwestern-most portions of India, in freshwater and coastal habitats [9]. Miocene fossils from *Anhinga* are known from adjacent Pakistan [10], and although one cormorant specimen was reported in the Siwalik Hills of India [2], their close relatives, the darters, have not previously been described from India. However, the taxonomic and phylogenetic status of the Siwalik Hills cormorant specimen needs verification because of the similarities of skeletal elements among cormorants and anhingids (Phalacrocoracoidea) and given the fossil specimen’s original identification as a potential tropicbird (Phaethontidae) [11] (and see text below).

The taxonomy of the extant species level taxa of Anhingidae is divided into two opinions. One scenario groups all of the Old World individuals into a single species (*Anhinga melanogaster*) with three subspecies, and a single New World species (*A*. *anhinga*) comprising two subspecies (see [12] for a historical review). The alternative taxonomy supports the occurrence of four species (*A*. *anhinga* in the New World, *A*. *rufa* in Africa and part of the Middle East, *A*. *melanogaster* in Asia, and *A*. *novaehollandiae* in Australasia) [12]. However, there is some slight variation in that taxonomy present in the proposal by Dorst and Mougin [13], where they group the Australasian and African members into a single subspecies, *A*. *melanogaster rufa*. Attempts at taxonomic and phylogenetic resolution based on morphological data [14,15] have provided data conflicting with those traditional taxonomies as well. Part of the reason for these contrasting approaches to the species level taxonomy is the result of the overall morphological similarity of the extant anhingids, but the genetic distance data of Christidis and Boles [16] supports species level rank for all four geographic/continental groups. For the purposes of this paper, we will follow the two species (with three Old World subspecies) concept consistent with the boundaries used by Worthy [17] in his recent assessment of a fossil anhingid.

There are many known Neogene fossils of darters in the Old World, and they include specimens from the Miocene of Thailand [18], the Miocene of Kenya [19,20], the Miocene of Ethiopia and Chad [21], the Miocene of Hungary [22], the Miocene of Tunisia [23], the Pliocene of Ethiopia [24], the Pliocene of Libya [25], and the Neogene (and Pleistocene) of Australia [17,26,27]. Lambrecht’s [22] taxon *Anhinga pannonica* (from eastern Europe) has had material from Africa [21,23], Pakistan [10], and elsewhere allocated to it [17,27]. The fossil femur from the Miocene of Japan originally published as a cormorant [28] was identified as an anhingid by Rich et al. [29], but there are no characters or analyses presented to support its position with Anhingidae as opposed to Phalacrocoracidae. In addition, there are many Neogene anhingid taxa described from the New World, including very large and small sized species (e.g., [15,30]).

Despite that seemingly broad assemblage, there appears to be only three or four published records of anhingids from Asia. There are specimens referred to *Anhinga pannonica* from the late Miocene of Pakistan [10], *Anhinga* cf. *pannonica* from the Miocene of Thailand [18], *Anhinga* sp. from the late Miocene of Abu Dhabi [31], and the possible anhingid from the Miocene of Japan [28,29]. All of those fossils are Miocene in age, and the specimen described below appears to be the first Pliocene anhingid from Asia. The Pakistan material referred to *A*. *pannonica* [10] includes part of a tarsometatarsus, and the specimens from Thailand include a proximal tarsometatarsus [18], but the remaining sites with fossil specimens of *Anhinga* in Asia have not produced additional tarsometatarsi. Furthermore, the Miocene specimens from Kenya, Tunisia, Chad, and Ethiopia do not include a tarsometatarsus [20,21,23], and a tarsometatarsus is absent from the material described as *Anhinga hadarensis* from the Pliocene of Ethiopia [24]. Additionally, the Pliocene anhingid from Australia [27] also lacks material from the foot, although its smaller (‘pygmy’) body size likely precludes it from being conspecific with this new material. That situation limits the level of comparison that can be made with the new Indian fossils (that comprise different portions of the foot).

Institutional/collection abbreviations: KP/KK/BS–Khetpurali/Kewal Krishan/Bahadur Singh, housed in the Department of Anthropology, Panjab University, India; MVZ–Museum of Vertebrate Zoology, University of California, Berkeley, USA; UCMP–University of California Museum of Paleontology, Berkeley, USA; VPL/RP-KPB–Vertebrate Palaeontology Laboratory (Geology Department, Panjab University, India)/Rajeev Patnaik-Khetpurali Bird.

Osteological terminology follows Baumel and Witmer [32] with some English equivalents of the Latin terms used. The terminology for the canals and grooves of the hypotarsus follows Mayr [33]. No permits were required for the described study, which complied with all relevant regulations. No specific permissions were required for field collection of fossils because this is an academic geological study and moreover, the overall project is funded by the Ministry of Earth Science, Government of India. Fossil collection in the field and museum comparative work did not involve any living animals, only fossils and skeletons housed in museum collections.

### Comparative material examined

Anhingidae

*Anhinga anhinga leucogaster* MVZ 85509

*Anhinga melanogaster novaehollandiae* MVZ 143017

*Anhinga melanogaster novaehollandiae* (*papua* race) MVZ 149268

Phalacrocoracidae

*Phalacrocorax auritus auritus* MVZ 151575

*Phalacrocorax capillatus* MVZ 124049

*Phalacrocorax carbo novaehollandiae* MVZ 143257

*Phalacrocorax melanoleucos melanoleucos* MVZ 156698

*Phalacrocorax gaimardi* MVZ 157988

*Phalacrocorax harrisi* MVZ 140913

*Phalacrocorax brasilianus mexicanus* MVZ 46167

*Phalacrocorax pelagicus resplendens* MVZ 19089

*Phalacrocorax penicillatus* MVZ 46809

*Phalacrocorax sulcirostris sulcirostris* MVZ 143250

*Phalacrocorax punctatus punctatus* MVZ 164582

*Phalacrocorax varius hypoleucos* MVZ 143267

*Phalacrocorax urile* MVZ 154268

Sulidae

*Morus bassanus* UCMP 131057

*Morus* sp. UCMP 123175 and 137455

*Sula leucogaster* MVZ 54774

*Sula sula* MVZ 132906

## Systematic paleontology

Order PELECANIFORMES Sharpe, 1891 [34]

Family ANHINGIDAE Lesson, 1831 [35]

Genus *ANHINGA* Brisson, 1760 [36]

*ANHINGA* sp.

### Referred specimens

**Fig 2:**
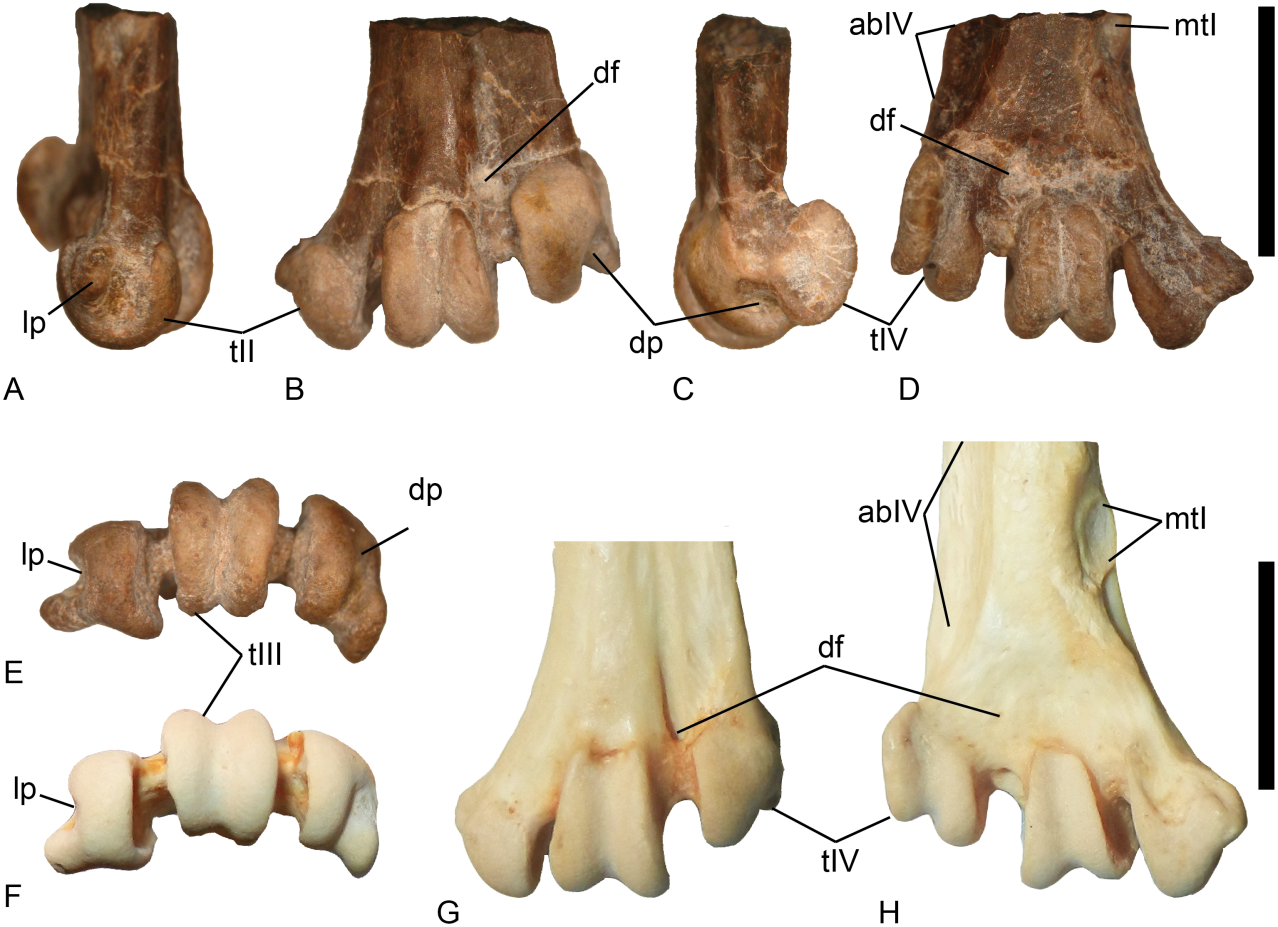
Fossil and recent anhingid distal tarsometatarsi. *Anhinga* sp. (VPL/RP-KPB1) from India in: (A) Medial view; (B) Dorsal View; (C) Lateral View; (D) Plantar view; and (E) Distal view. *Anhinga melanogaster* (MVZ 149268) in: (F) Distal view; (G) Dorsal view; and (H) Plantar view. Scale bars equal 1 cm. The specimens are presented at the same size, but the *Anhinga melanogaster* specimen is larger (bottom scale bar) as compared to the fossil (top scale bar). Abbreviations: abIV–groove for m. abductor digiti IV; df–distal vascular foramen; dp–deep pit on the distal face of metatarsal trochlea IV; lp–collateral ligament pit; mtI–facet for metatarsal I; tIII–metatarsal trochlea III; and tIV–metatarsal trochlea IV.

**Fig 3:**
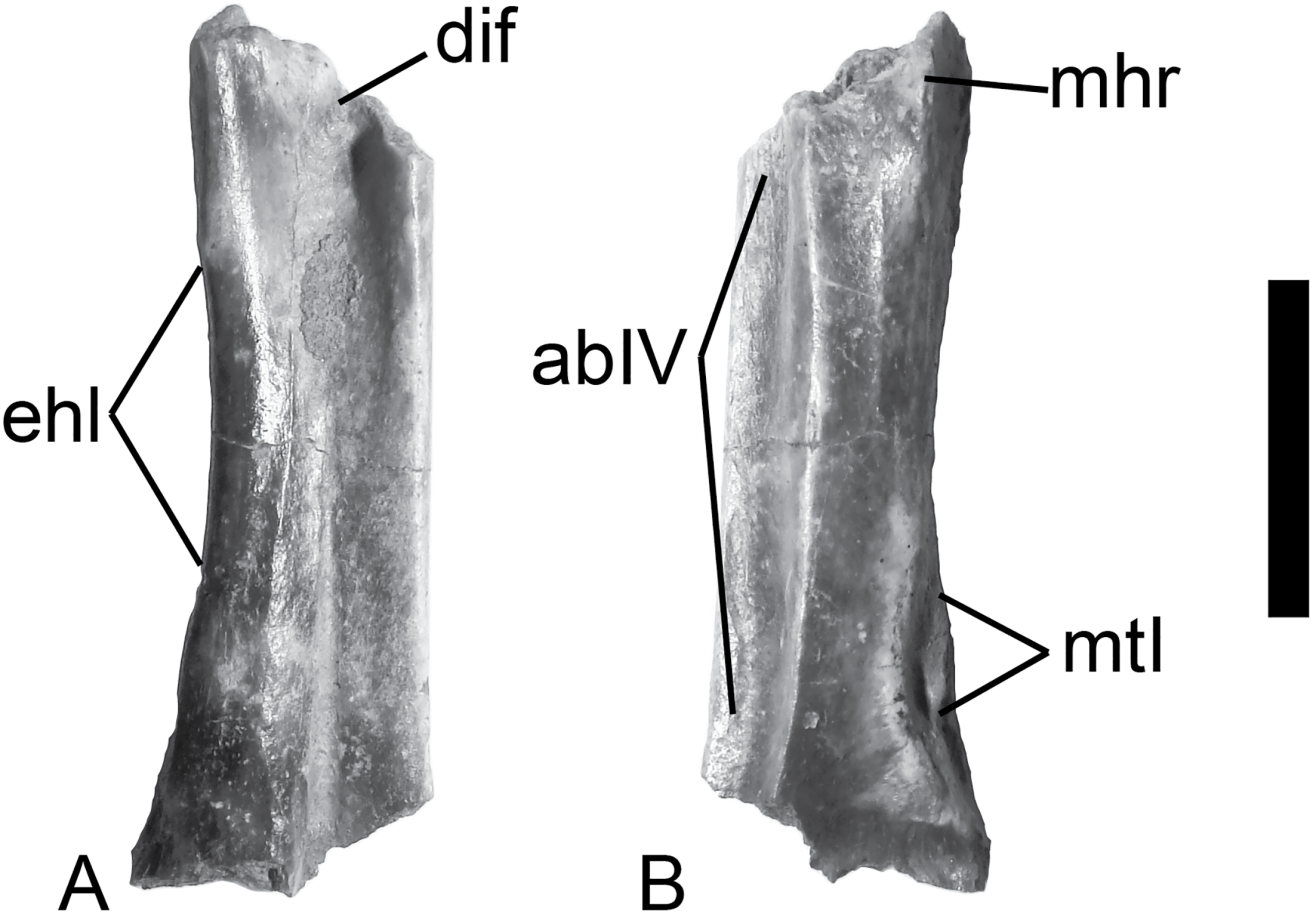
The *Anhinga* tarsometatarsus shaft (KP/KK/BS/102) from the Khetpurali section, India. (A) Dorsal view. (B) Plantar view. Scale bar equals 1 cm. Abbreviations: abIV–m. abductor digiti IV groove; dif–dorsal infracotylar fossa; ehl–broad notch for the m. extensor hallucis longus; mhc–medial hypotarsal crest; and mtI–metatarsal I facet.

**Fig 4:**
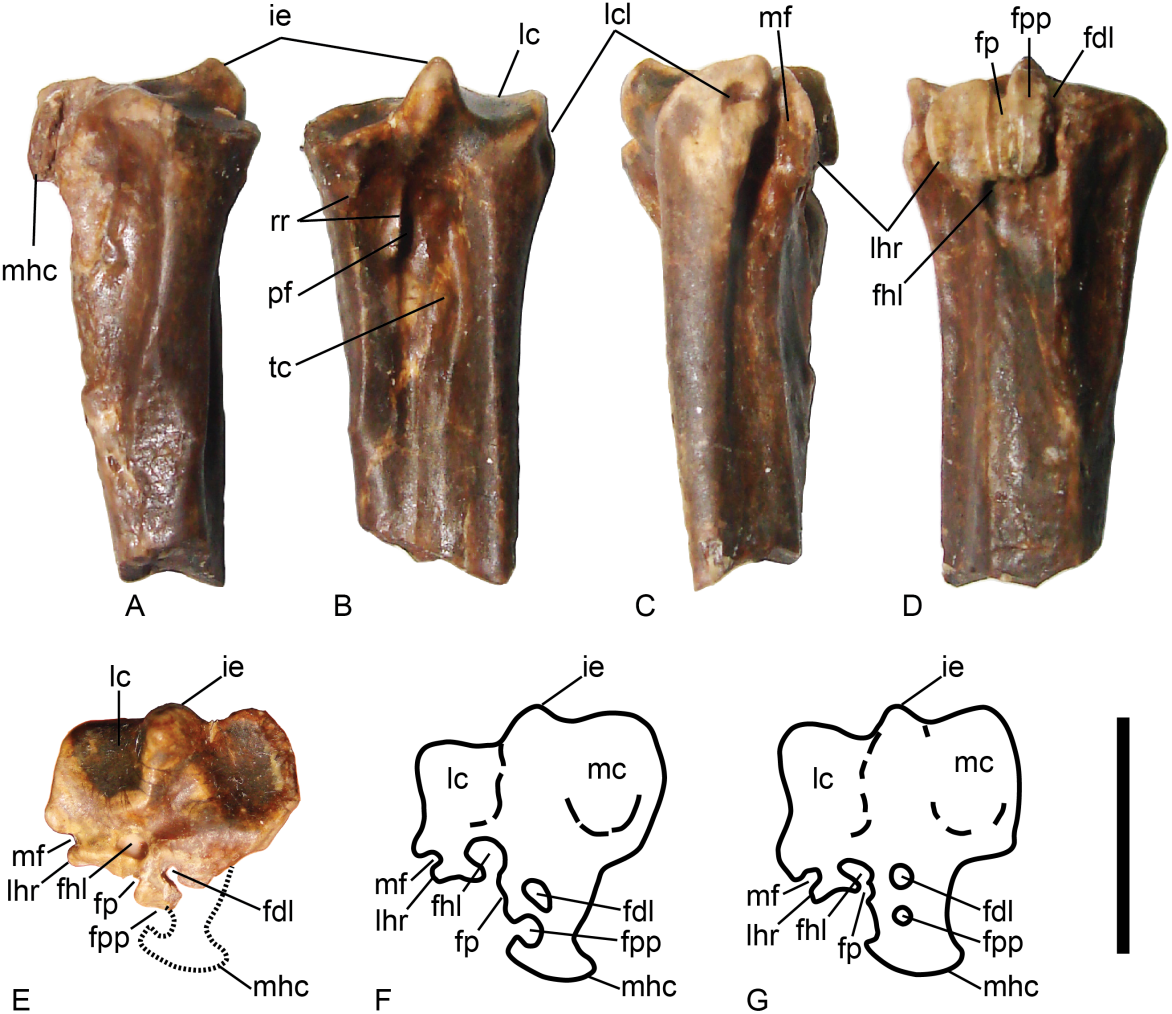
The proximal tarsometatarsus of fossil and extant Anhingidae with a focus on the hypotarsus. The Siwalik Hills specimen (KP/KK/BS/101) in: (A) Medial view; (B) Dorsal view; (C) Lateral view; (D) Plantar view; and (E) Proximal view (the missing medial hypotarsal crest is marked by a dashed line). Outline of the tarsometatarsus of *Anhinga melanogaster* in: (F) Proximal view. Outline of the tarsometatarsus of *Anhinga anhinga* in: (G) Proximal view. Outline drawings redrawn from Harrison’s [37] type A and B morphologies. Scale bar equals 1 cm. Abbreviations: fdl–canal for the m. flexor digitorum longus tendon; fhl–canal for the m. flexor hallucis longus tendon; fp–groove for the m. flexor perforatus digiti II tendon; fpp–groove/canal for the m. flexor perforans et preforatus digiti II tendon; ie–intercotylar eminence; lc–lateral cotyle; lcl–impression for the lateral collateral ligament; lhr–lateral hypotarsal ridge (ridge bounding the groove for the m. fibularis longus tendon); mc–medial cotyle; mf–groove for the m. fibularis tendon; mhc–medial hypotarsal crest; pf–proximal vascular foramen; rr- retinacular ridges; and tc–attachment for the m. tibialis cranialis.

VPL/RP-KPB1 is a distal left tarsometatarsus (Fig 2), KP/KK/BS/102 is a partial left tarsometatarsus missing the proximal and distal ends (Fig 3), and KP/KK/BS/101 is a proximal left tarsometatarsus (Fig 4). The fossils derive from three different individuals since two specimens preserve the distal part of the metatarsal I facet (i.e., overlapping morphology), the mid shaft specimen overlaps in its morphology with the proximal fragment (sharing the distal end of the medial hypotarsal crest), and all specimens are from the left side. All three (osteologically adult) specimens likely derive from the same species given their near identical sizes, corresponding overlapping morphologies, co-occurrence in the same fossil locality, and the presence of a combination of anhingid synapomorphies and plesiomorphies in all specimens (see below).

### Locality and age

The specimens were collected from a bone-bearing horizon exposed in the upper part of the Tatrot Formation (Khetpurali Section) in the Siwalik Hills of Northern India that is latest Pliocene in age (~2.6 Ma), and the locality produced a previously published pelican specimen (see [4]; Fig 1). Detailed locality information is available to qualified researchers.

### Diagnostic features

On the plantar side, the shaft specimen KP/KK/BS/102 (Fig 3) exhibits a distinct groove for the m. abductor digiti IV and a distinct (though worn) notch for the m. extensor hallucis longus that are a synapomorphies of Phalacrocoracoidea (Phalacrocoracidae + Anhingidae; [17]). The plantar opening of the distal vascular foramen is obscured, but clearly smaller than the dorsal opening, also considered diagnostic for anhingids within Phalacrocoracoidea [17]. The proximal Khetpurali specimen KP/KK/BS/101 exhibits tendinal canals for the m. flexor digitorum longus and m. flexor hallucis longus having roughly equal diameters, with the canal for the m. flexor digitorum longus positioned only slightly plantar to the level of the m. flexor hallucis longus (Fig 4), and Worthy [17] considered that morphology as a unique anhingid character. The fossils also exhibit features present in anhingids and considered plesiomorphic among pelecaniforms [17] including the distal tarsometatarsus specimen displaying a shortened metatarsal trochlea IV (with the mediolateral width being greater than the proximodistal length of the trochlea distal to the proximal edge of the lateral intertrochlear incisure), and a metatarsal trochlea IV in line with the shaft of the tarsometatarsus (Fig 2). The combination of these derived and primitive features (and others described below) uniquely occur among anhingids.

The estimated length of the tarsometatarsus is approximately 46 mm, and it is longer than that of *A*. *anhinga*, shorter than that of *A*. *walterbolesi*, and within the size range of *A*. *melanogaster* (comparative length data from table 1 in [17]). The size of the distal end also is consistent with the extant species (Table 1). The hypotarsus morphology of the Indian specimen shares the laterally open groove for the m. flexor perforans et perforatus digiti II tendon with all subspecies groups placed in *A*. *melanogaster* (the Type B morphology of Harrison [37]). However, there are morphological differences between the Siwalik Hills anhingid specimens and other extinct and extant specimens. The Siwalik Hills specimens exhibit a greatly enlarged medial plantar flange (in distal view) of metatarsal trochlea II (with a rather concave plantar surface) (Fig 2E) that extends distinctly beyond the dorsal rim of the collateral ligament pit (similar to the state in the much older *A*. *walterbolesi*), a proximal plantar edge of the lateral trochlear rim of metatarsal trochlea IV that forms a hook with a concave proximal margin (absent in *A*. *walterbolesi*), a very pointed intercotylar eminence (as compared to the blunter shape in extant and extinct anhingids), and a distinct very deep pit on the distal face of metatarsal trochlea IV (Fig 2) that is an enlargement of the trochlear furrow (absent in the extant taxa and not described for any extinct anhingid taxon). Thus, it is possible that these fossils represent an early member of the *A*. *melanogaster* group, but allocation to other extinct species cannot be ruled out with this material.

**Table 1:** Distal tarsometatarsus measurements (mediolateral width) of fossil and extant anhingids (in mm).

Species	Distal width	Reference/specimen
*Anhinga* sp.	14.5	VPL/RP-KPB1
*Anhinga anhinga*	13.7–14.6	[17]
*Anhinga anhinga leucogaster*	13.8/13.7	MVZ 85509
*Anhinga melanogaster*	14.9–17.4	[17]
*Anhinga m*. *novaehollandiae*	16.5	MVZ 143017
*Anhinga m*. *novaehollandiae*	15.9/16.0	MVZ 149268
*Anhinga walterbolesi*	18.1	[17]

### Description

The preserved portions of the tarsometatarsus (from all three specimens) derive from a tarsometatarsus that would have been approximately 46 mm long. That length was derived from a measurement of the total length of the three specimens when their overlapping morphologies are placed in line. The description below proceeds from the distal specimen through the midshaft specimen, to the proximal end fragment.

Specimen VPL/RP-KPB1 preserves the most distal portion of a left tarsometatarsus and is broken at its proximal end through the metatarsal I facet leaving only its distal tip preserved (Fig 2). The lateral side of the plantar face of the specimen is concave to the base of metatarsal trochlea IV (though narrowing distally), and the lateral edge of the bone is very thin (dorsoplantarly). The area just proximal to metatarsal trochlea III on the plantar surface is slightly concave, including the area around the plantar opening of the distal vascular foramen. The preserved plantar shaft of the tarsometatarsus is relatively flat (proximal to the concave area). The plantar opening of the distal vascular foramen is obscured and its exact size is not clear, but it is reduced compared to the dorsal opening.

Metatarsal trochlea II extends distal to trochlea III, and that extends distal to trochlea IV. The lateral edge of the bone is flush with the lateral surface of metatarsal trochlea IV (dorsal view) meaning that metatarsal trochlea IV is in line with the tarsometatarsal shaft (not laterally displaced). In contrast, metatarsal trochlea II is distinctly projected mediodistally with the medial margin of the bone being concave (dorsal/plantar view). Metatarsal trochlea II is asymmetrical with a single large bulbous portion dorsally and a concave plantar face. The medial face of metatarsal trochlea II has a large collateral ligament pit that extends proximally making a notch in the proximal medial edge of the trochlea. In addition, there is a very large plantar medial flange on metatarsal trochlea II that extends far medially with its apex near the proximodistal midpoint of the trochlea, and the apex is slightly worn. The plantar edge of metatarsal trochlea II also is positioned plantar to the other trochleae. The lateral edge of metatarsal trochlea II is concave, and the lateral edge continues proximally (plantar face) as a raised ridge that ends at the same proximodistal level as the apex of the medial plantar flange. The intertrochlear incisions are approximately equal in width, but the lateral incision extends more proximally (and is rather short). Metatarsal trochlea III has a furrow extending from its proximal dorsal end to its proximal plantar end. The area just proximal to the dorsal proximal end of metatarsal trochlea III is slightly concave, and the proximal end of the plantar surface of the trochlea is rounded and symmetrical. The medial and lateral sides of metatarsal trochlea III are nearly flat. Metatarsal trochlea IV is asymmetric with a large plantar flange and a deep pit on the distal face. The lateral surface of metatarsal trochlea IV has a collateral ligament pit in the plantar half of that face (deepens dorsally), and there is a smaller concave area dorsal to the collateral ligament pit (near the dorsoplantar level of the dorsal end of the large pit on the distal face). The proximal edge of the lateral flange of metatarsal trochlea IV is concave with a distinct notch. The pit on the distal face is deepest dorsally and extends plantarly (shallowing) as a concave area on the plantar face of the trochlea. The dorsal surface of metatarsal trochlea IV is flat to slightly convex. The dorsal part of the medial face of metatarsal trochlea IV is concave with the dorsal medial edge of the trochlear surface overhanging the intertrochlear space (distal to the distal vascular foramen). There is a tendinal groove proximal to the dorsal opening of the distal vascular foramen. Lateral to that groove, the dorsal face of the shaft is relatively flat and more plantar in relation to the area medial to the groove that is convex over its surface.

Specimen KP/KK/BS/102 preserves most of the mid-shaft of the tarsometatarsus from the metatarsal I facet proximally to the distal end of the hypotarsus, and it is 27.0 mm long (Fig 3). The facet for metatarsal I sits within a broader concave area on the medial plantar face of the shaft, and the proximal end of the facet is quite close to the proximodistal level of the distal end of the broad notch for the m. extensor hallucis longus. There is a shallow, but distinct tendinal groove proximal to the distal vascular foramen that extends proximally to just distal to the proximodistal midpoint of the specimen. The notch for m. extensor hallucis longus is proximodistally longer than the mediolateral width of the shaft, and the proximal and distal ends of the notch are rounded. Overall, it appears that the shaft fragment is worn. The proximal dorsal end preserves part of the dorsal infracotylar fossa and what appears to be one of the rugosities for the m. tibialis cranialis (medial side). The central portion of the plantar face of the shaft is a distinct flat face that separates the laterally concave area from the convex medial face. The lateral edge of the shaft has a thin edge to it. The distal base of the medial hypotarsal crest is preserved at the proximal end.

The tarsometatarsus fragment KP/KK/BS/101 is the proximal portion broken near the distal end of the dorsal infracotylar fossa, and the medial hypotarsal crest is mostly missing (Fig 4). The specimen is 22.2 mm long, and the proximal end is 10.9 mm wide (mediolaterally). The intercotylar eminence projects proximally and ends in a narrow point. The medial cotyle has a rim around most of its edge, and the dorsal edge is positioned distal to the plantar edge (it is angled distally). The lateral cotyle lacks a rim around its dorsal edge, and that dorsal edge curves onto the dorsal face of the bone. There is a small concavity plantar to the intercotylar eminence and in between the cotyles. The medial edge of the lateral cotyle is proximal to the level of the lateral edge of the medial cotyle. The proximal lateral corner of the tarsometatarsus has two pits with the smaller deeper pit plantar and more proximal as compared to the shallower pit. In proximal view, the lateral edge of the lateral cotyle is nearly straight and directed at a near right angle with the dorsal edge of the cotyle.

The dorsal infracotylar fossa is deep proximally, and the lateral rim of the fossa extends distal to the medial rim. The proximal vascular foramina are near the medial and lateral edges of the dorsal infracotylar fossa and close to the same proximodistal level as the ridges for the attachment of the extensor retinaculum (on the medial rim of the extensor fossa). The pair of ridges for the extensor retinaculum appear to be highly asymmetrical with the lateral one forming the thin proximal medial edge of the dorsal infracotylar fossa (dorsal to the medial proximal vascular foramen), and the medial one seems to be a short more proximally positioned ridge. However, the area distal to the preserved medial attachment of the extensor retinaculum is damaged, and the ridge may have extended more distally. The area between the two retinacular attachments is concave. The medial rim of the extensor retinaculum distal to the broken area near the retinacular attachment is concave medially, and the proximal end of the broad notch for the m. extensor hallucis longus muscle is preserved near its distal end. The medial margin of the bone just proximal to the proximal end of the broad notch for the m. extensor hallucis longus is shallowly concave. There is a distinct lateral tuberosity for the m. tibialis cranialis near the mediolateral midpoint of the extensor fossa distal to the proximal vascular foramina, and a thin distally elongate ridge in the more medial portion of the fossa. The medial tuberosity for the m. tibialis cranialis is as distinct as the lateral one.

The medial plantar face of the tarsometatarsus is a shallow (parahypotarsal) fossa that extends distally slightly less distal as compared to the medial rim of the extensor fossa. The plantar opening of the medial proximal vascular foramen is near the proximodistal midpoint of that fossa. The lateral edge of that fossa is formed by a (damaged) ridge that extends distally from the medial hypotarsal crest. The area distal to the two main preserved tendinal canals (of the hypotarsus) in the central portion of the plantar tarsometatarsus shaft is slightly concave over its surface and raised above the adjacent medial and lateral faces. The lateral face of the plantar shaft is a proximodistally elongate fossa for m. abductor digiti IV (that extends onto the other specimens described above) adjacent to the thin lateral edge of the bone. The lateral proximal vascular foramen opens plantarly near the border between the fossa for the m. abductor digiti IV and the central raised area, and the two proximal foramina are at approximately the same proximodistal level.

There is no large plantar ridge (medial crest) of the hypotarsus preserved (present in phalacorcoracoids, Fig 4), but it appears to have been broken away with a broken base extending distally. Overall, the preserved hypotarsus is relatively short (proximodistally). The large canal for the m. flexor hallucis longus is plantar to the lateral cotyle, and it is fully enclosed (not a plantarly open groove). The plantar face of the hypotarsus plantar to the m. flexor hallucis longus canal is distinctly concave with its lateral edge forming a ridge that demarcates the groove for the m. fibularis longus at the lateral plantar corner of the hypotarsus. The m. flexor digitorum longus canal is positioned plantar to the intercotylar eminence and slightly plantar to the dorsoplantar level of the m. flexor hallucis longus canal. The m. flexor digitorum longus canal is damaged with it open medially (related to the loss of the medial hypotarsal crest), but likely it was fully enclosed before the breakage of the bone. The preserved most plantar portion of the hypotarsus (in between the m. flexor digitorum longus and m. flexor hallucis longus canals and plantar to them) preserves a shallow groove that likely is the open groove for the tendon of the m. flexor perforans et perforatus digiti II (figure 3M in Mayr’s [33] application of terminology), or for the tendons of the m. flexor perforatus digiti II and m. perforans et perforatus digiti II (figure 2F in Worthy’s [17] interpretation of the same tendinal groove). There does not appear to be missing bone in the fossil specimen lateral to that tendinal groove for digit II, and likely it was open laterally in life. There is a separate deep groove immediately plantar to the m. flexor hallucis longus canal that presumably is for the m. flexor perforatus digiti II (Fig 4). In plantar view, the lateral edge of the hypotarsus (in particular the flange bordering the m. fibularis longus groove at the lateral edge of the bone) is semicircular in outline with the convexity directed laterally.

### Comparisons

It appears that the metatarsal I facet is overall more distally positioned than the state in *Anhinga* cf. *pannonica* from the Miocene of Pakistan [10] and *Anhinga walterbolesi* [17]. The figure relating to the anhingid specimen from Pakistan apparently is mislabeled with the fossils and the modern specimen reversed (figure 1 in [10]). In those images (figure 1 in [10]), it appears that the facet for metatarsal I is proximally closer to the broad notch for the m. extensor hallucis longus in the Pakistani specimen referred to *A*. cf. *pannonica* than the state in *A*. *anhinga*. The proximal edge of the facet in the Indian specimen is at the same level as the distal end of the notch unlike that illustrated for *A*. *anhinga*, but more similar to that shown for the Pakistani specimen (figure 1 in [10]).

The anhingid tarsometatarsus from the Miocene of Thailand reportedly has the laterally open groove for the m. flexor perforans et perforatus digiti II tendon (as in *A*. *melanogaster* and the Indian specimen above), but its intercotylar eminence is rounded, in contrast to the pointed condition in the Siwalik Hills fossil. The Khetpurali proximal tarsometatarsus also has a narrower proximal mediolateral width (11.0 mm) than that of the *A*. cf. *pannonica* specimen from Thailand (12.4 mm) and *A*. *grandis* (13.8 mm) from the Miocene of the USA [18].

The Indian specimens differ from the Oligo-Miocene *Anhinga walterbolesi* in several ways. The Khetpurali material deviates from *A*. *walterbolesi* in having a flattened lateral edge of metatarsal trochlea IV in distal view, as compared to the state in *A*. *walterbolesi* with the trochlear furrow forming a notch in the lateral edge (figure 2 in [17]). In addition, the Indian specimens are smaller (15.0 mm mediolateral distal width) than *A*. *walterbolesi* (18.1 mm; [17]; Table 1). The feature of an open groove for the m. flexor digitorum longus tendon present in *A*. *walterbolesi* is absent (fully enclosed) in the Indian specimen. Additionally, the impression of the lateral collateral ligament just distal to the lateral cotyle projects more laterally than the state in the Indian specimen (figure 2 in [17]; Fig 4). The dorsal portion of the medial face of metatarsal trochlea IV dorsal to the lateral intertrochlear incision in *A*. *walterbolesi* reportedly does not have an overhanging flange [17]. There is a slight concavity in that face in the Indian specimen, but lacks a distinct flange. The Indian specimen shares with darters and non-crown cormorants the state of having the proximodistal length of the broad notch in the medial edge of the shaft for the m. extensor hallucis longus being greater than the mediolateral width of the shaft [17]. While the plantar medial process of metatarsal trochlea II is large in both the Indian specimen and *A*. *walterbolesi*, it projects more laterally (and is dorsal to the plantar edge of the lateral trochlear rim) than the more medioplantarly directed process in *A*. *walterbolesi*. *Anhinga walterbolesi* also lacks the notch in the proximal plantar lateral rim of metatarsal trochlea IV (lateral view), and the enlarged pit on the distal face of metatarsal trochlea IV (figure 2 in [17]). The great age disparity between the Indian material (late Pliocene) and *A*. *walterbolesi* (Oligo-Miocene) further precludes the likelihood of them being conspecific.

The tarsometatarsus is not known from Pliocene African *Anhinga hadarensis* [24], and thus cannot be directly compared to the Indian material. However, the reported distal width of the tibiotarsus for *A*. *hadarensis* is 10.0 mm, close in size to that of extant species [24]. With the proximal mediolateral width of the Indian tarsometatarsus being 11.0 mm, the Indian individuals should be within the size range to potentially correspond to the tibiotarsus of *A*. *hadarensis*.

There are many extinct species of anhingas in South America that range broadly in size with many of the species much larger than the Indian specimen and some species not known from a tarsometatarsus [17,30]. Those fossils have not been reported to have the greatly enlarged plantar projection of metatarsal trochlea II or the unique metatarsal trochlea IV morphology [30]. They also have more rounded intercotylar eminences than that of the Indian specimen [15,30]. The hypotarsus of *Macranhinga* and *Meganhinga* have an enclosed tendinal canal for m. flexor perforans et perforatus digiti II set within the medial hypotarsal crest, consistent with the state in *A*. *anhinga* (figure 1 in [15]). That state differs from that of the open groove for that tendon in the Indian specimen and reportedly in all extant members of Old World darters [37]. Therefore that feature may represent a character (apomorphy) uniting the extant and extinct New World anhingid taxa because it also is absent among extant cormorants [33] and the early stem phalacrocoracid *Nambashag* [38]. If that character is a synapomorphy of New World anhingids (including the Neogene fossil species), then its phylogenetic distribution would suggest that the divergence between the Old World and New World species lineages occurred during the Miocene, and further may support the species level recognition of the individual Old World groups. The Indian specimens are within the size range of the extant species (Table 1), but are smaller than *A*. *walterbolesi* and most of the South American (giant) fossil taxa [17].

The Khetpurali specimens differ from the extant material examined as well, while sharing some features with *A*. *melanogaster* such as the laterally open tendinal groove for the m. flexor perforans et perforatus digiti II. Metatarsal trochlea II extends distal to trochlea III (which is distal to trochlea IV) in *Anhinga anhinga leucogaster* (MVZ 85509) and *Anhinga melanogaster novaehollandiae* (MVZ 143017 and 149268; Fig 2). That state is not present among the cormorant skeletons examined (where metatarsal trochlea II is equal to trochlea III or more proximal) and appears that it may be a synapomorphy of (or within) the anhingid lineage since it is absent in sulids and frigatebirds (the next two outgroups beyond cormorants). Another character that appears to differentiate cormorant and anhinga tarsometatarsi is the presence of a concave plantar face of metatarsal trochlea II in all fossil and extant anhingids examined (flat plantar face in all cormorants examined; Fig 2). Both Mayr [33] and Harrison [37] illustrate the canal for the m. flexor hallucis longus tendon as being open (i.e., a groove and not an enclosed canal) in their specimens of *Anhinga*, similar to that in cormorants. However, all MVZ specimens of both *Anhinga* species examined (see above) have an at least partially enclosed canal for that tendon, demonstrating variation in that character that is perhaps ontogenetic. The enclosed canal also is present in the phalacorcoracoid outgroup Sulidae (e.g., *Morus bassanus* UCMP 131057) [33].

There is a relatively large plantar medial projection from metatarsal trochlea II in *Anhinga anhinga leucogaster* and *A*. *melanogaster novaehollandiae*, similar to, but perhaps not quite as pronounced as in the Khetpurali material. The size of that projection varies among the cormorants examined with it larger in some species (e.g., *P*. *brasilianus mexicanus*) and smaller in others (e.g., *P*. *pencillatus*), but that feature does not approach the extremely large state in the Indian fossil or that in extant anhingas (Fig 2). The dorsoplantar position of metatarsal trochlea II is similar to the state in the fossil in the extant anhingas examined (although it looks like the position in *A*. *anhinga* is a bit more dorsal). In all cormorants examined (except *Phalacrocorax gaimardi* and *P*. *brasilianus mexicanus*, and *P*. *pelagicus resplendens*), metatarsal trochlea II is more plantar than the state in the fossil and extant anhingids. The plantar proximal end of metatarsal trochlea III is relatively wide and symmetrical in the fossil. It shares that state with *Anhinga anhinga leucogaster*, and differs from the asymmetrical and narrowed proximal end in *P*. *carbo novaehollandiae*, *P*. *capillatus*, *P*. *auritus auritus*, *P*. *melanoleucus melanoleucus*, *P*. *gairmardi*, *P*. *brasilianus mexicanus*, *P*. *punctatus punctatus*, and *P*. *urile*. In *P*. *harrisi*, the proximal end narrows, but is rounded, the state in *P*. *penicillatus*, *P*. *varius hypoleucos* and *P*. *sulcirostris sulcirostris* is narrow and symmetric, and the state is more similar to the fossil in *P*. *pelagicus resplendens*. *Anhinga melanogaster* varies a bit from a broad, but slightly asymmetric end, to a narrowed, but symmetrical shape. The fossil displays a notch in the proximal plantar base of metatarsal trochlea IV (lateral view). That notch is absent in *A*. *anhinga leucogaster*, *A*. *melanogaster novaehollandiae* (but it has a concave proximal margin), and all cormorants examined, but one specimen of *A*. *melanogaster novaehollandiae* (MVZ 149268) has a slightly broader notch than the state in the fossil.

It would appear based on the comparisons available that these new Asian anhingid specimens might represent a new species. The material is younger (latest Pliocene versus Miocene) than the other specimens from Asia. At a minimum, they appear to differ from the material referred to *A*. *pannonica* and from the extant species. The Indian material cannot be directly compared to *A*. *hadarensis* from the Pliocene of Ethiopia [24] because there is no tarsometatarsus known for that species, but they are of similar age (and likely size, see above). Given the wide (intercontinental) geographic distributions of the extant species of *Anhinga* (Old World and New World taxa), it is possible that the African (Hadar) and Indian (Khetpurali) specimens could be conspecific. Furthermore, the features of the Indian fossil taxon (i.e., the greatly enlarged medial plantar projection from metatarsal trochlea II, the laterally open groove for the m. flexor perforans et perforatus digiti II tendon, and pointed intercotylar eminence) might have a greater variability within species than currently realized, and that potential variability may mean that these specimens could be conspecific with a known extinct taxon (that is presently known from other skeletal elements) or represents an extinct lineage within *A*. *melanogaster*. It also is possible that these fossils could represent an anagenetic lineage leading to one of the extant species/subspecies, but that cannot be determined until a larger sample of specimens is found. It should be noted that specimens allocated to *Anhinga melanogaster* have been published from the Miocene, Pliocene, and Pleistocene of Africa suggesting the antiquity of the species lineage [39], but no anhingid tarsometatarsi are known from those localities that would allow for comparison.

The proximal tarsometatarsus identified as a cormorant from the Siwalik Hills [2] originally was presented as a potential tropicbird relative [11]. Lydekker [2] suggested morphological similarity between it and *Phalacrocorax carbo*, but admitted that he did not do extensive comparisons. In the illustration of the fossil [2], it can be seen that the cormorant specimen differs from the new Indian material in the presence of a more rounded intercotylar prominence that does not project far proximally (as compared to the more sharply pointed and tall projection in the new material). The cormorant specimen is broken through what appears to be the proximal end of the notch for the m. extensor hallucis longus, and features of the hypotarsus are not clearly described or illustrated. Therefore, the relative size of that medial notch and details of the tendinal canals and grooves of the hypotarsus cannot be assessed accurately. It is hard to interpret, but it appears that the cormorant was drawn with an open groove for the tendon of the m. flexor hallucis longus that would contrast with the closed canal in the new Indian specimen. None of the features provided by Lydekker [2] or visible in his illustrations of the specimen clearly diagnose that fossil as a cormorant, as opposed to an anhingid (such as *A*. *pannonica*). This cormorant specimen from an unknown Neogene horizon in the Siwalik Hills sequence needs to be reexamined in order to determine its phylogenetic affinities, though it most likely is distinctly older (i.e., Miocene) than the Kherpurali material (latest Pliocene).

## Discussion

The presence of a pelican and multiple anhingid individuals at the same locality/stratigraphic horizon in the Khetpurali section of the Siwalik Hills all point to a strong aquatic influence on the site that is today over 1000 km from the nearest part of the Indian Ocean. The associated fauna of this bone-bearing horizon in the Tatrot Formation includes crabs, crocodiles, and fish, in addition to the avian fossils [4,40], and the depositional environment initially was interpreted as a floodplain pond. The fossils derive from paleosols associated (stratigraphically above and below) with medium grained sandstones typical of floodplain deposits, suggestive of the prevalence of aquatic environments around the fossil site. The discovery of several aquatic bird specimens deriving from multiple individuals in the horizon would seem to indicate that the site/fossil horizon might have been more than a mere pond, and perhaps a lake or at least a relatively larger body of water with a variety of water depths. Furthermore, these avian taxa (pelicans and darters) are piscivorous and point to the past abundance of fish in the area. Extant anhingids prefer open, but not very deep water, and occur from coastal areas to interior wetlands with projections (vegetation or rocks) present above water that allow the birds get out of the water in order to dry their wings [12]. The presence of multiple individuals of an anhingid clearly supports the past presence of that type of habitat in the foothills of the Himalayan Mountains during the latest Pliocene.

Similar to the pelican taxon from the same locality, the anhingid specimens cannot be placed clearly into any extant species (though *A*. *anhinga* can be excluded as a possibility), although fossils allocated to *A*. *melanogaster* are known from older and younger sediments in Africa [39]. These Khetpurali anhingid specimens also are younger than other records in Asia (allocated to the extinct European species *A*. *pannonica*), likely deriving from a new taxon or lineage (potentially diagnosed by the extremely large medial projection of metatarsal trochlea II, the morphology of metatarsal trochlea IV, and extremely pointed intercotylar eminence) that may be an extinct part of the *A*. *melanogaster* grouping of Old World individuals. Other workers have supported the evolution of the *A*. *melanogaster* group by the Miocene in Africa, but early representatives of that group have not yet been reported in Asia (see [39] for a listing of publications). If the pelican fossil is added to this discussion, its phylogenetic placement (like the anhingid) potentially outside of extant species lineages (but associated with an Old World clade) is suggestive that (aquatic) birds from the latest Pliocene of India may represent an extinct avifauna that was replaced by the extant species lineages during the Pleistocene (currently missing from the fossil record in the region). The mammalian fossil record from the Siwalik Hills suggests a turnover event at approximately 2.58 Ma [41] slightly younger than the age estimate for this avian locality (~2.6 Ma), and close to the Neogene-Quaternary Boundary there was a major faunal turnover with a large number of African and European mammalian taxa dispersing into India during the early Pleistocene [42]. Given the ecological or environmental ramifications of such a drastic faunal change and wave of immigrants, perhaps the avian fauna also experienced a replacement in biodiversity in the earliest Pleistocene (possibly from taxa that immigrated to India), but only future finds will help to resolve the timing and pattern of the origin of the modern Indian avifauna. Nevertheless, these fossils demonstrate the presence of darters in southern Asia around the time of this transition from Neogene to Quaternary faunas.
